# Communicator-Driven Data Preprocessing Improves Deep Transfer Learning of Histopathological Prediction of Pancreatic Ductal Adenocarcinoma

**DOI:** 10.3390/cancers14081964

**Published:** 2022-04-13

**Authors:** Raphael M. Kronberg, Lena Haeberle, Melanie Pfaus, Haifeng C. Xu, Karina S. Krings, Martin Schlensog, Tilman Rau, Aleksandra A. Pandyra, Karl S. Lang, Irene Esposito, Philipp A. Lang

**Affiliations:** 1 Department of Molecular Medicine II, Medical Faculty, Heinrich-Heine-University, Universitätsstrasse 1, 40225 Düsseldorf, Germany; raphael.kronberg@hhu.de (R.M.K.); melanie.pfaus@hhu.de (M.P.); xuh@uni-duesseldorf.de (H.C.X.); kakri104@uni-duesseldorf.de (K.S.K.); 2 Mathematical Modelling of Biological Systems, Heinrich-Heine University, Universitätsstrasse 1, 40225 Düsseldorf, Germany; 3 Institute of Pathology, Medical Faculty, Heinrich-Heine University and University Hospital of Duesseldorf, Moorenstr. 5, 40225 Düsseldorf, Germany; lenajulia.haeberle@med.uni-duesseldorf.de (L.H.); martin.schlensog@med.uni-duesseldorf.de (M.S.); Tilman.Rau@med.uni-duesseldorf.de (T.R.); irene.esposito@med.uni-duesseldorf.de (I.E.); 4 Department of Pediatric Oncology, Hematology and Clinical Immunology, Medical Faculty, Center of Child and Adolescent Health, Heinrich-Heine-University, Moorenstrasse 5, 40225 Düsseldorf, Germany; aleksandra.pandyra@uni-duesseldorf.de; 5 Institute of Immunology, Medical Faculty, University of Duisburg-Essen, Hufelandstr. 55, 45147 Essen, Germany; KarlSebastian.Lang@uk-essen.de

**Keywords:** computer vision, deep learning, metastases, pancreatic cancer, pancreatic ductal adenocarcinoma, transfer learning

## Abstract

**Simple Summary:**

Pancreatic cancer has a dismal prognosis and its diagnosis can be challenging. Histopathological slides can be digitalized and their analysis can then be supported by computer algorithms. For this purpose, computer algorithms (neural networks) need to be trained to detect the desired tissue type (e.g., pancreatic cancer). However, raw training data often contain many different tissue types. Here we show a preprocessing step using two communicators that sort unfitting tissue tiles into a new dataset class. Using the improved dataset neural networks distinguished pancreatic cancer from other tissue types on digitalized histopathological slides including lymph node metastases.

**Abstract:**

Pancreatic cancer is a fatal malignancy with poor prognosis and limited treatment options. Early detection in primary and secondary locations is critical, but fraught with challenges. While digital pathology can assist with the classification of histopathological images, the training of such networks always relies on a ground truth, which is frequently compromised as tissue sections contain several types of tissue entities. Here we show that pancreatic cancer can be detected on hematoxylin and eosin (H&E) sections by convolutional neural networks using deep transfer learning. To improve the ground truth, we describe a preprocessing data clean-up process using two communicators that were generated through existing and new datasets. Specifically, the communicators moved image tiles containing adipose tissue and background to a new data class. Hence, the original dataset exhibited improved labeling and, consequently, a higher ground truth accuracy. Deep transfer learning of a ResNet18 network resulted in a five-class accuracy of about 94% on test data images. The network was validated with independent tissue sections composed of healthy pancreatic tissue, pancreatic ductal adenocarcinoma, and pancreatic cancer lymph node metastases. The screening of different models and hyperparameter fine tuning were performed to optimize the performance with the independent tissue sections. Taken together, we introduce a step of data preprocessing via communicators as a means of improving the ground truth during deep transfer learning and hyperparameter tuning to identify pancreatic ductal adenocarcinoma primary tumors and metastases in histological tissue sections.

## 1. Introduction

In histopathological diagnostics, malignant neoplasms are detected and classified based on the analysis of microscopic tissue slides stained with hematoxylin and eosin (H&E) under a bright-field microscope. A precise classification of malignant neoplasms is pivotal for adequate patient stratification and therapy. In some cases, a histopathological diagnosis can be challenging, even when ancillary techniques for tissue characterization, such as immunohistochemistry (IH) or molecular analyses, are applied. Pancreatic ductal adenocarcinoma (PDAC) is a highly aggressive cancer type arising from the epithelial cells of the pancreatobiliary system. PDAC is usually recognized at an advanced stage [1] when it has already metastasized to the lymph nodes, peritoneum, liver or lungs [1,2]. Surgical resection is currently the only curative therapy for patients with PDAC. However, as the majority of patients present with locally advanced disease or distant metastases, there is a lack of effective treatment options [2,3]. In patients undergoing surgery, a definitive diagnosis of PDAC is achieved by a histopathological evaluation of surgical resection specimens. If a patient is not eligible for surgery, diagnostic confirmation is reached through a histopathological assessment of biopsy samples obtained during an endosonographic ultrasonography.

Deep neural networks can be used for the classification of images. Specifically, convolutional neural networks are multilayered and trained with a back-propagation algorithm to classify shapes [4]. In medicine, convolutional neural networks are used to classify images to predict clinical parameters and outcomes [5,6]. Deep neural networks can also be used to identify histological patterns [7]. Studies have shown that tissue sections from non-small lung cancer can be classified and their mutational profile predicted using deep transfer learning [8]. Patient outcomes can also be predicted from histology images. This has been demonstrated in studies of colorectal cancer [9,10], as well as for hepatocellular carcinoma patients following liver resection [11]. RNA-Seq profiles and prognostic features, such as microsatellite instability, can also be predicted from slide images of gastrointestinal cancers [12,13]. Importantly, using deep transfer learning of the model inception v3 and The Cancer Genome Atlas image database, most cancer types can be predicted from histological images [14]. One issue with data preparation for deep learning is that histological images are composed of multiple tissue components. Some datasets divide a histological image into subgroups, such as adipose tissue, mucosa and lymphoid tissue [10]. Currently, although a variety of networks are used for histological classification, including AlexNet, DenseNet, ResNet18, ResNet50, SqueezeNet, VGG-16 and VGG-19 [15,16,17,18], it is challenging to find a network with the ability to effectively filter out confounding histological tissue entities.

Using a new dataset consisting of healthy pancreases, healthy lymph nodes and PDAC, we show that histological material can be purified using two communicating neural networks, which we termed “Communicators”. Based on an existing dataset, we added one class of our data which filtered the training data of Communicator 2. The purified dataset provided the training data for a convolutional neural network to classify these labels. The network was validated on further independent histological sections. Interestingly, the trained network was able to identify PDAC metastases in lymph nodes. Further, extensive hyperparameter testing suggests that the Resnet fine-tuned network with the ADAM Optimizer and a learning-rate of 0.0001 was efficient in this setting.

## 2. Materials and Methods

**Patient Data:** Histological images of PDAC and healthy pancreatic tissue were obtained from tissue micro arrays (TMAs) [19]. For the dataset, we used a cohort of well-characterized PDAC patients (*n* = 229). Two hundred and twenty-three PDAC tissue spots (one per patient) and 161 healthy pancreas tissue spots (one per patient) were used. A second anonymized TMA cohort contained healthy lymph node samples (*n* = 78), of which 76 spots were used (Supplementary Table S1). All tissue samples were obtained from patients who underwent surgical cancer resection at the University Hospital of Düsseldorf, Germany. Additionally, a third cohort contained whole-slide tissue images with different tissue types for validation. We used four evaluation sets with 10 patients: PDAC consisting of 15 images, healthy pancreas (HP) consisting of 3 images, lymph node (LN) with PDAC having 6 images, and healthy lymph node (HLN) with 5 images. To establish adequate ground truth for validation, the digitalized whole-slide images were annotated manually on the *regional level*, distinguishing healthy pancreas, normal lymphatic tissue, PDAC, adipose tissue and other “background tissues”, such as blood vessels [20].

**Tissue acquisition and preparation:** Tissue samples were acquired from the routine diagnostic archive of the Institute of Pathology, Düsseldorf, Germany. All tissue samples were fixed in 4% buffered formaldehyde and embedded in paraffin blocks. For the preparation of tissue microarrays (TMAs), samples with a 1-mm core size from primary tumors (PDAC), lymph node metastases and corresponding normal tissue were selected and assembled into the respective TMA (Manual Tissue Arrayer MTA-1, Beecher Instruments, Inc., Sun Prairie, WI, USA). Hematoxylin & eosin staining was prepared from 2-µm thick tissue sections of the TMA blocks and whole-slide tissue blocks according to the protocol established in the routine diagnostic laboratory of the Institute of Pathology of Düsseldorf, Düsseldorf, Germany.

### 2.1. Digitalization of H&E Tissue Slides

H&E tissue slides were digitalized using the Aperio AT2 microscopic slide scanner (Leica Biosystems, Wetzlar, Germany). H&E slides were scanned using either the 40× magnification (TMA slides) or the 20× magnification (whole-tissue slides). Microscopic image files were saved as Aperio ScanScope Virtual Slide (.SVS) files and displayed using Aperio ImageScope software 12.3.3 (Leica Biosystems, Wetzlar, Germany). Tissue spots were extracted from the TMAs using the Aperio Imagescope software. The images were resized to 50% of the pixel size with Image Resizer for Windows (version 3.1.1.) when scanned with a 40× magnification. In addition to tissue slides acquired as described above, we also obtained a previously described dataset composed of the following tissue type: adipose tissue (ADI, 10.407 images) [10].

### 2.2. Deep Transfer Learning

The preprocessing pipeline included a 50% zoom on Unpatched Images, and normalization [21]. Images were dissected into image tiles, fitting the input size of the neural networks.

**Architecture:** We used a deep transfer learning approach for the network architecture [22]. We chose to fine-tune and adapt the residual neural network Resnet18 [23], as previously described [24]. In addition to the transformations, we added a Gaussian Blur for training as augmentation. We retrained the last three layers of the Resnet18. Adam [25] was used as the optimizer for this deep transfer learning approach. A square image patch size of 224 pixels was used. We trained the network with the batch sizes of 150 and 100 epochs, early stopping of 5 on the images of 80% of the samples from the dataset using the pathologist’s label as ground truth. We balanced the dataset by random doubling of the images in the underrepresented classes. The predicted probability for each image patch to contain each of the labels (HLN, HP, PDAC, ADI, BG) was used as the objective/loss function (Cross Entropy Loss) in the training. We used an initial learning rate of 0.0001 and a decrease by 5% every five epochs. Evaluation was carried out by applying the previously trained model to the remaining, previously unseen 20% of the dataset for each sequence set separately and comparing the results with the ground truth. In addition to the accuracy, we calculated the confusion matrix, the precision, recall, Jaccard index and the F1-score for each class. We used early stopping, based on the loss of the validation learning, with early stopping equaling 5 [26]. For further evaluation, the algorithm classified the tissue type by patch labeling of separate validation images. For visualization, we colored each image patch in the color of the predicted class.

**Metrics:** For comparison and evaluation of our models, we used the following five metrics; metrics for the binary case are shown.

The scores of the metrics are in the Interval (0,1) and, therefore, the greater the score, the better.

$$Precision=\frac{TP}{TP+FP}$$

$$Recall=\frac{TP}{TP+FN}$$

$$F1-Score=\frac{2TP}{2TP+FP+FN}$$

$$Jaccard-Score=\frac{TP}{TP+FP+FN}$$

$$Accuracy=\frac{TP+TN}{TP+FP+TN+FN}$$

For the multiclass (non-binary) case, the positive is the target class and the other classes are the negative class. With this definition, separate metrics were obtained for *TP*, *FP*, *TN* and *FN*.

**Classification score vector:** A classification score summing up the classification labels of each patch of an image and pointing to the percentual portion of this class was determined.

$$c:\;=\left(c_{1},\ldots ,c_{i},\ldots ,c_{N}\right),$$

where *i* ∈ 1, …, *N* and *N* is the number of classes. With the definition of the patch vector

$$p:\;=\left(p_{1},\ldots ,p_{j},\ldots ,p_{M}\right),$$

where *j* ∈ 1,…,M and *M* is the number of patches for this image. Then we defined

$$c_{i}:=\frac{\sum_{j=1}^{M}1_{f\left(p_{j}\right)=i}}{\sum_{j=1}^{M}1},$$

where *f* is the prediction function of the neural network. The dominator guarantees that the sum of the vector entries is equal to one. We used the classification vectors to determine the image label by argmax of the patch labels, if not otherwise stated.

**Three score:** For a second score to rank the networks, we calculated the percentages of the right label prediction. The average of image tiles of a group (HLN, HP, PDAC) on the test data was determined.

**Four score:** A segmentation tool was used to rank different networks on the validation dataset, by a pathologist [27]. The average of image tiles of a group (HLN, HP, PDAC and LNPM) was determined and compared to the pathologist’s label as ground truth. Images with insufficient labeling were excluded, as indicated.

The Four score is defined by

$$fourscore=1-\frac{1}{4}\sum_{1}^{4}m_{i}$$

where the m_i_s are given by

$$m_{i}=\mathrm{abs}\left(\sum_{j=1}^{N_{i}}c_{i}^{\left(j\right)}-p_{i}\right),$$

where the *c_i_*^(*j*)^ is ith entry of the classification vector for the jth extern validation image, and *p_i_* is the average over all images of one class prediction, annotated by the pathologists. The m_is are called HLN-score, HP-score, PDAC-score and LNPM-score.

### 2.3. Software & Hardware

Training and validation was performed on a Nvidia A100 of the high performance cluster (HPC, Hilbert) of the HHU, and on Quadro T2000 with Max-Q Design (Nvidia Corp., Santa Clara, CA, USA), depending on the computational power needed.

On the workstation, we used the Python VERSION:3.8.8 [MSC v.1916 64 bit (AMD64)] software (pyTorch VERSION:1.9.0.dev20210423, CUDNN VERSION:8005). On the high-performance cluster we used the following software: Python VERSION:3.6.5 [GCC Intel(R)\\ C++ gcc 4.8.5 mode] (including pyTorch VERSION:1.8.0.dev20201102+cu110, CUDNN VERSION:8004).

## 3. Results

### 3.1. Communicating Neural Networks Enrich New Datasets for Parenchymal Tissue

**Figure 1:**
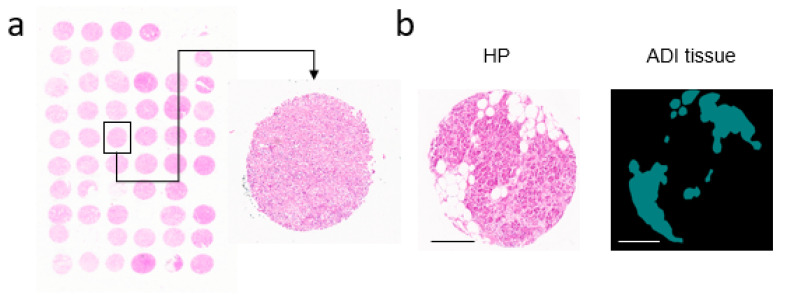

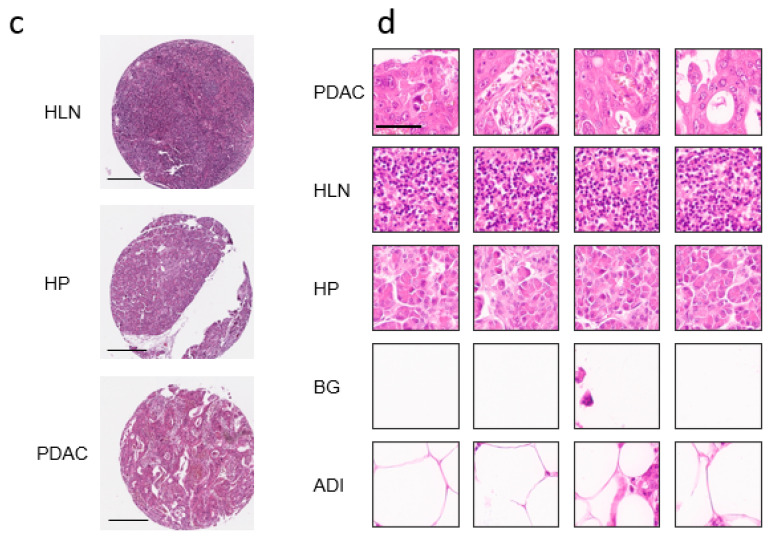
Data Pre-processing Pipeline for H&E-stained Tissue Micro Arrays provide datasets for Deep Transfer Learning. (**a**) Patients’ data were extracted from tissue micro arrays (TMAs) and annotated. (**b**) A representative HP Spot with adipose tissue and a segmentation of the adipose tissue are shown (scale bar = 300 µm). (**c**) Spots from healthy lymph node (HLN), healthy pancreas (HP) and pancreatic ductal adenocarcinoma (PDAC) (scale bar = 300 µm). (**d**) Whole images were cut into square patches with 224 × 224 pixel sizes (scale bar = 60 µm). PDAC, HLN, HP, Background (BG), and Adipose Tissue (ADI) sample image tiles are shown.

To investigate whether PDAC can be detected by convolutional neural networks, we obtained histological images of healthy pancreatic tissue, healthy lymph node (HLN) tissue and pancreatic ductal adenocarcinoma (PDAC) tissue. Each tissue section was extracted from scanned images of tumor microarrays (TMAs) for further data preprocessing (Figure 1a, Supplementary Figure S1). However, tissue samples and, consequently, histological images did not contain only image tiles attributed to their respective label. Specifically, adipose tissue was observed in some images (Figure 1b). Furthermore, artefacts could be observed in tissue images from TMAs (Figure 1c). Accordingly, when tissue sections were dissected into 224 × 224-pixel image squares to match the size of the input layer of the convolutional neural network ResNet18 [24], the image tiles showed a variety of tissue identities, including adipose tissue and background, which did not match the respective label (Figure 1d). Overall, we obtained 17,842 image patches for HP, 9954 patches for HLN tissue and 25,650 patches for PDAC. We therefore speculated that the ground truth was not ideal in this setting, necessitating further data preprocessing.

**Figure 2:**
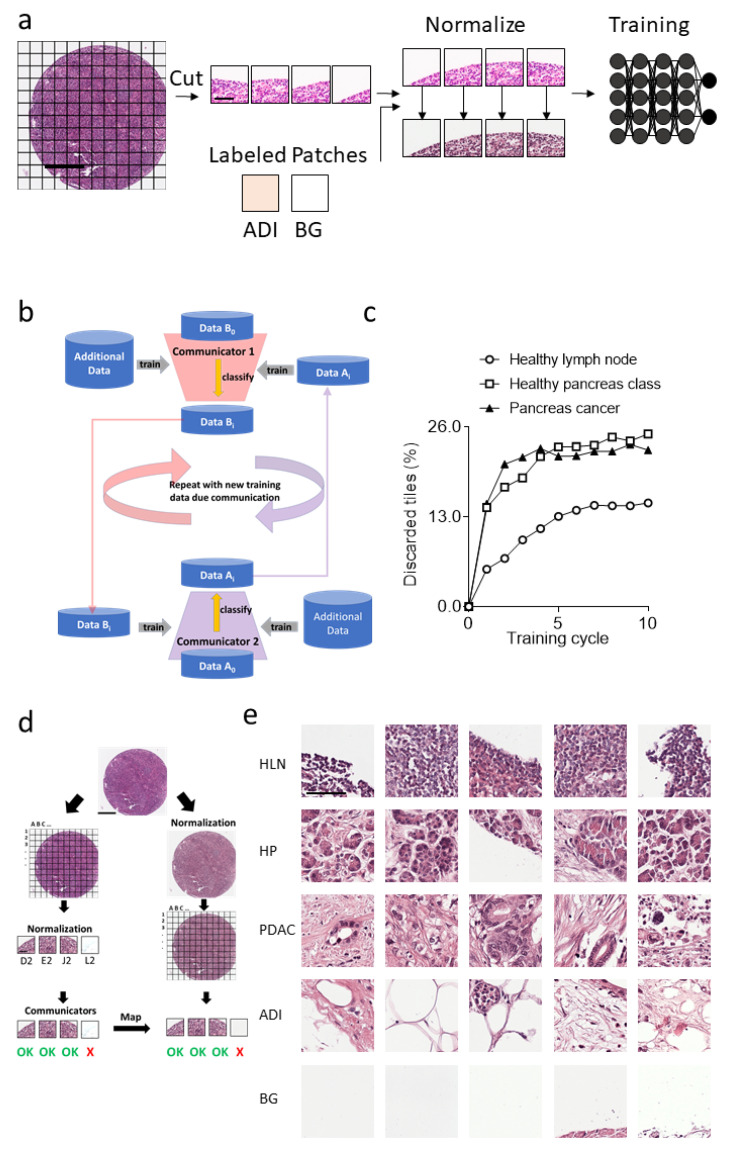
Data clean-up via communicators improves ground truth by introducing more labels. (**a**) The schematic view of preprocessing and training of the CNNs (Spot: Scale bar = 300 μm, Patch: Scale bar = 60 μm). (**b**) Schematic set up of the communicators used for data clean-up. (**c**) Percentage of discarded image patches of the different tissue types during the clean-up process from healthy lymph nodes, healthy pancreas and pancreatic ductal adenocarcinoma is indicated. (**d**) Selection of the normalized tissue patches based on the classification of the communicator CNNs is illustrated (Spot: Scale bar = 300 μm, Patch: Scale bar = 60 μm). (**e**) Representative communicators sorted tissue patches from three cleaned-up tissue classes and the two extracted new classes are shown (scale bar = 60 µm). Tissue patches of healthy lymph nodes (HLN), healthy pancreases (HP), pancreatic ductal adenocarcinoma (PDAC), background (BG), and adipose tissue (ADI) labels are presented.

To purify the image tiles within each label, we made use of deep transfer learning on the ImageNet database’s pretrained network, ResNet18 [22,23]. Specifically, an existing dataset containing labeled image tiles of adipose tissue was associated with tiles from 20 images of a new dataset class labeled Data A_i_ [10,28]. Since tissue sections were obtained from different image slides, we normalized the H&E staining intensity on image tiles, as previously described (Figure 2a) [21]. This dataset was used to train Communicator 1, which then removed image tiles from 20 different images of the new dataset class that were not classified as the new dataset class, resulting in a dataset labeled Data B_i_ (i-th iteration of the process) (Figure 2b). The selected Data B_i_ image dataset was used along with the existing dataset for the training of Communicator 2 (Figure 2b). Communicator 2 removed confounding images from the dataset Data A_i_ images, resulting in an improved dataset Data A_i+1_ (Figure 2b). This process was repeated through several cycles, i, to remove other tissue types, such as adipose tissue from the new datasets. Using this process, we reduced the number of tiles for the labels and purified the ground truth (Figure 2c). Notably, other network architectures, such as VGG11 or Densenet, can also be used for communicator-based purification of dataset classes (Supplementary Figure S2). The final Resnet18 communicators were used to remove all image tiles that were not classifiable on all input data images with a threshold of 0.55 on the softmax output. Since processing via the communicators relied on the normalization of image tiles to make use of a labeled dataset, we mapped image tiles to tiles generated from images which were normalized in toto (Figure 2d). Image tiles related to tiles the communicators labeled as adipose tissue or background were moved into a new dataset class (Figure 2d). Accordingly, the clean-up process through the communicators resulted in 13,261 image tiles for the healthy pancreas, 19,313 image tiles for PDAC, 8264 image tiles for HLN, 9952 images tiles for BG and 1235 image tiles for ADI (Figure 2e). Notably, the tissue patches selected by the communicators were not homogeneous as, for instance, the class label PDAC also included cancer-associated stromata, and inflamed/necrotic tissue (Figure 2e).

### 3.2. Dataset Clean-Up Improves Performance during Image Recognition

**Figure 3:**
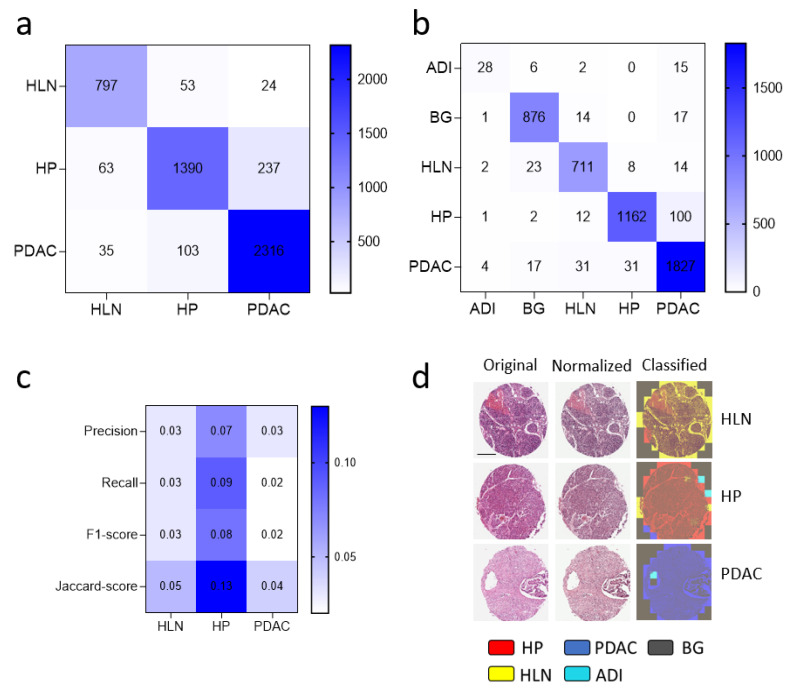
Data clean-up using communicators improves network’s performance. Confusion Matrices from retraining the neural network with (**a**) original test data or (**b**) with data post-clean-up via communicators are shown. (**c**) Heatmap of the performance difference between the network trained on data with and without the data clean-up via the communicators is shown. (**d**) Representative test spots from healthy lymph nodes (HLN), healthy pancreases (HP) and pancreatic ductal adenocarcinoma (PDAC) classified with the neural network are shown (scale bar = 300 µm). HLN (yellow), HP (red), PDAC (blue), background (BG, grey) and adipose tissue (ADI, cyan) were predicted by the retrained CNN.

Next, we used the obtained image tiles for the retraining of a convolutional neural network. Hence, the patient cohort was divided into training (80%), validation (10%), and test (10%) datasets. The image tiles in the different dataset groups were taken from different patients. Deep transfer learning was performed in retraining the last 3 blocks (18 layers) of the network ResNet18 using a learning rate of 0.0001, Adam loss function, and an early stopping of 5, as previously described [24]. The neural network trained on the raw dataset ((test: 1690, train: 14,450, val: 1702) image patches for healthy pancreases, (874, 8275, 805) patches for HLN tissue, (2454, 20,694, 2502) patches for PDAC) achieved a weighted accuracy over all classes of 90%, a weighted Jaccard score of 81% and a weighted F1-score of 90% (Figure 3a, Table 1). For the single classes, the F1-score was 86% (HP) and 92% (PDAC). The Jaccard score was 82% (HLN) and 85% (PDAC) (Figure 3a, Table 1).

When we used the purified image data training set ((test: 1277, train: 10,767, val: 1217) image tiles for healthy pancreases, (1910, 15, 605, 1798) image tiles for PDAC, (758, 6848, 668) image tiles for HLN, (908, 7971, 908) image tiles for BG and (51, 1049, 135) image tiles for ADI), we observed an improvement in the confusion matrix (Figure 3b). Specifically, the neural network showed an increased performance for the HP class of the recall of 9% (up to 91%), a Jaccard score of 13% (88%) and F1-score of 8% (94%) (Figure 3c, Table 1 and Table 2, Supplementary Tables S2 and S3). In addition, we visualized the patch-class labels in the tissue sections from the test dataset (Figure 3d). Notably, when we used the communicators for only 3 data clean-up cycles, we still observed an improved performance (Supplementary Table S3, Supplementary Figure S3). These data indicate that the neural network based on ResNet18 could be retrained to classify PDAC from images of the H&E slide sections. Furthermore, the performance was improved by dataset preprocessing involving two communicators that purified parenchymal image tiles.

### 3.3. Convolutional Neural Networks (CNN) Classification of Histological Images of Primary Tumors and Lymph Node Metastases Can Be Improved through Hyperparameter Tuning during Training and Classification

**Figure 4:**
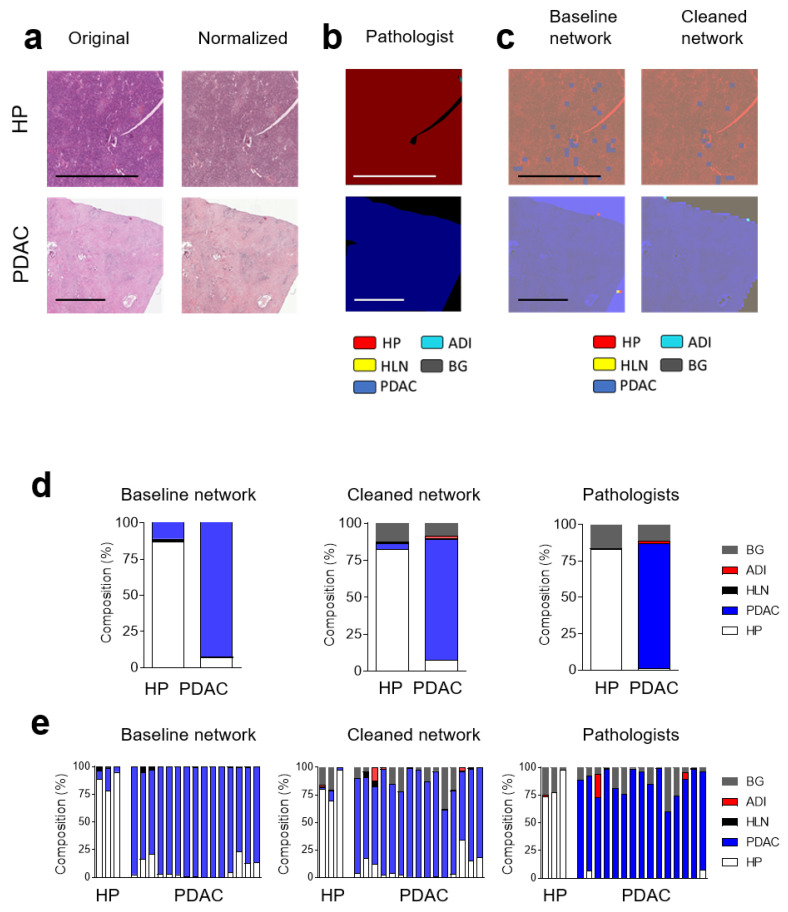
Convolutional Neural Network can classify healthy pancreas tissue and pancreatic ductal adenocarcinoma. (**a**) Sections from independent H&E-stained whole images from healthy pancreases (HP) and pancreatic ductal adenocarcinoma (PDAC) are shown (scale bar = 2 mm). (**b**) Expert label (ground truth), as determined by a pathologist and (**c**) classified with the baseline and cleaned network, are shown. HLN (yellow), HP (red), PDAC (blue), background (BG, grey) and adipose tissue (ADI, cyan) are indicated by the pathologist (**b**) or the CNNs (**c**). The pooled (**d**) and individual classification (**e**), as determined using a baseline and cleaned network, as well as by a pathologist, of whole-image slides from healthy pancreases (HP) (*n* = 3) and pancreatic ductal adenocarcinoma (PDAC) (*n* = 15) are shown.

To validate the retrained ResNet18, we used tissue sections of healthy pancreatic and PDAC tissue. Each image was normalized and divided into image tiles, which were classified according to the training labels (Figure 4a). The ground truth of this cohort was established by a pathologist, who labeled the histological images (Figure 4b). We noted that the majority of image tiles of histologically healthy pancreas tissue were labeled correctly. PDAC images were also correctly classified (Figure 4c–e). However, we also observed other classes appearing in healthy pancreas images (Figure 4c–e). This confusion likely resulted from other labels, including background, being present in pancreatic tissue that were not fed into the communicators.

**Figure 5:**
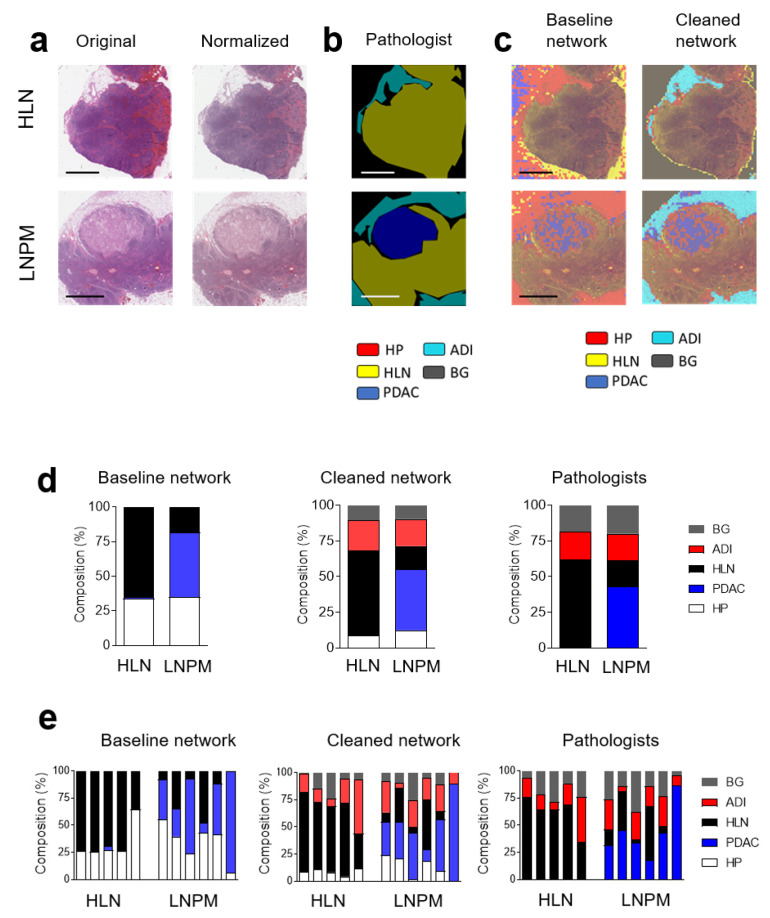
Convolutional Neural Network can classify metastases from pancreatic ductal adenocarcinoma in lymph nodes. (**a**) Sections from H&E-stained whole images from healthy lymph nodes (HLN) and lymph nodes with pancreatic ductal adenocarcinoma metastases (LNPM) are shown (scale bar = 2 mm). (**b**) Expert label (ground truth) as determined by a pathologist and (**c**) classified with the baseline and cleaned network are shown. HLN (yellow), HP (red), PDAC (blue), background (BG, grey) and adipose tissue (ADI, cyan) are indicated by the pathologist (**b**) or the CNNs (**c**). The pooled (**d**) and individual classification (**e**), as determined using a baseline and cleaned network, as well as by a pathologist, of whole-image slides from healthy lymph nodes (HLN) (*n* = 5) and lymph nodes with pancreatic ductal adenocarcinoma metastases (LNPM) (*n* = 6) are shown (scale bar = 2 mm).

To further validate our findings, we classified images from healthy and PDAC metastatic lymph nodes (Figure 5a). To compare different CNNs, a pathologist labeled images from different tissue types (Figure 5b). Following normalization, the images were labeled by the network trained with the cleaned or uncleaned dataset (Figure 5c). As expected, following the dataset clean-up, labeling by the CNN better reflected the labeling done by the pathologist (Figure 5c). Although the HLN was detected, we found a considerable amount of misclassified image tiles (Figure 5d). However, when we analyzed these images using the CNN trained with the purified dataset, the labeling improved significantly (Figure 5d). Furthermore, in image data from PDAC metastatic lymph nodes, a proportion of image tiles was classified as PDAC (Figure 5d,e). Notably, a substantial amount of misclassified tiles in the baseline model was due to background that was not eliminated during the data preprocessing. To evaluate whether the communicators demonstrated a beneficial effect, we removed background tiles with a pixel cutoff at 239, thereby removing most of the image tiles (Supplementary Figure S4a). However, when we purified the dataset after the pixel cutoff via the communicators, we still found improved labeling with the cleaned-up network (Supplementary Table S3, Supplementary Figure S4b–d). These data show that the retrained ResNet18 can detect PDAC in primary tumors (Supplementary Figure S5) and lymph node metastases and that the data clean-up process via communicators improved the labeling of histological images.

**Figure 6:**
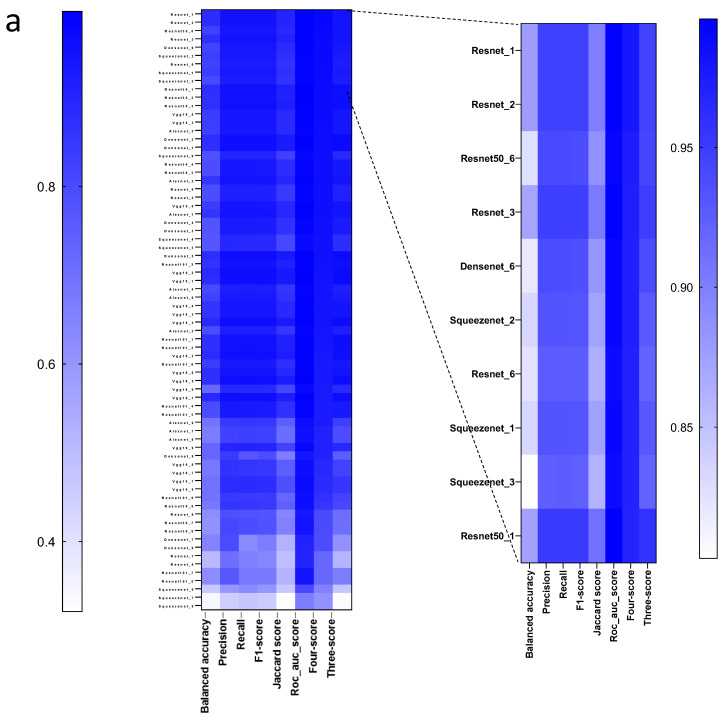

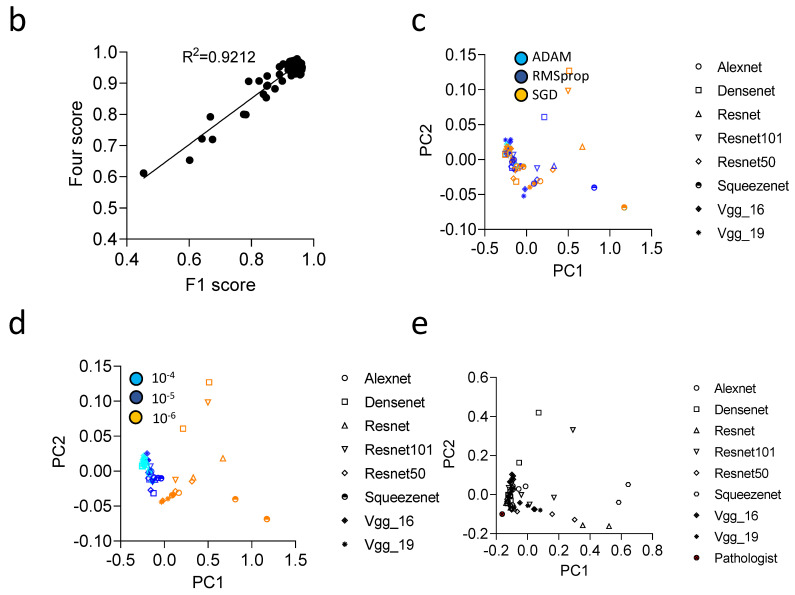
Hyperparameter Tuning illustrates the performance of different network architectures trained with variable learning rates and optimizers. (**a**) Performance of 72 trained and validated neuronal networks were ranked regarding the four-score, highlighting the best 10 network configurations. (**b**) Correlations were tested between F1-score and four-score via r2-score. (**c**,**d**) PCA (linear kernel) of the network metrics from hyperparameter tuning colored by the (**c**) different optimizers and architectures, (**d**) learning rates and architectures, and (**e**) PCA (linear kernel) of the modified four-score parts: HLN-score, HP-score, PDAC-score and LNMP-score (vs. the pathologist annotations over all 29 validation images).

To investigate whether different models or hyperparameters affected the CNNs’ performance, we trained 72 networks based on different network architectures, including ResNet18 [23], ResNet50 [23], ResNet101 [23], Vgg-16 [29], Vgg-19 [29], Alexnet [30], DenseNet [31] and SqueezeNet [32]. We also performed the training using different learning rates (ranging from 10^−4^ to 10^−6^) and optimizers (SGD, Adam [25], RMSprop). We evaluated the accuracy, Jaccard Score, F1-Score, and the classification of HP tissue, PDAC, HLN tissue and PDAC metastatic lymph nodes on independent images. The results of the networks were compared to the ground truth based on labeling by a pathologist (Figure 4b and Figure 5b, Supplementary Table S4). As expected, the networks showed a wide variety of performances dependent on the different training parameters (Figure 6a). The best performance in this setting was seen in the Resnet_1 network, which had a four-score of 97.8% of the pathologist’s labeling (Figure 6a, Supplementary Table S4). We observed a clear correlation between the performance on the test dataset and the independent validation dataset (Figure 6b). The different model architectures achieved a better performance with different optimizers (Figure 6c). While all network architectures were able to classify the validation images (Figure 6d), a clear dependence of the performance was associated with the learning rate (Figure 6d). Notably, a learning rate of 10^−6^ was not preferable in this setting compared to the other values (Figure 6d). Different models demonstrated different performances, and the gap to the annotated labels from the pathologist shows the performance as measured by the components of the four-score (Figure 6e). Taken together, these data indicate that dataset preprocessing, image classification stratification, and hyperparameter tuning can have an impact on the recognition of PDAC in lymph node tissue from H&E images.

### 3.4. Communicator Based Preprocessing Can Be Transferred to Other Input Sizes

**Figure 7:**
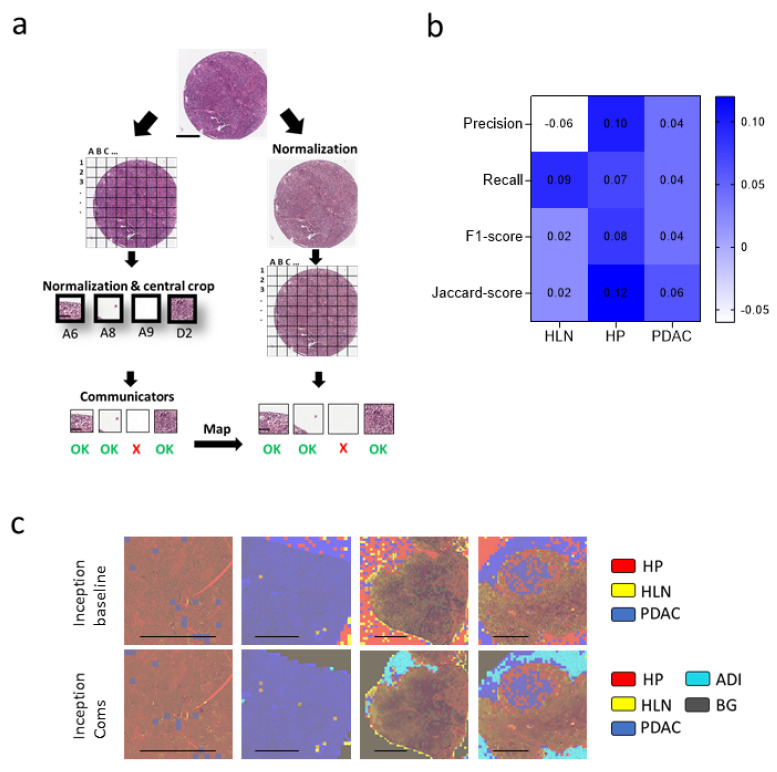

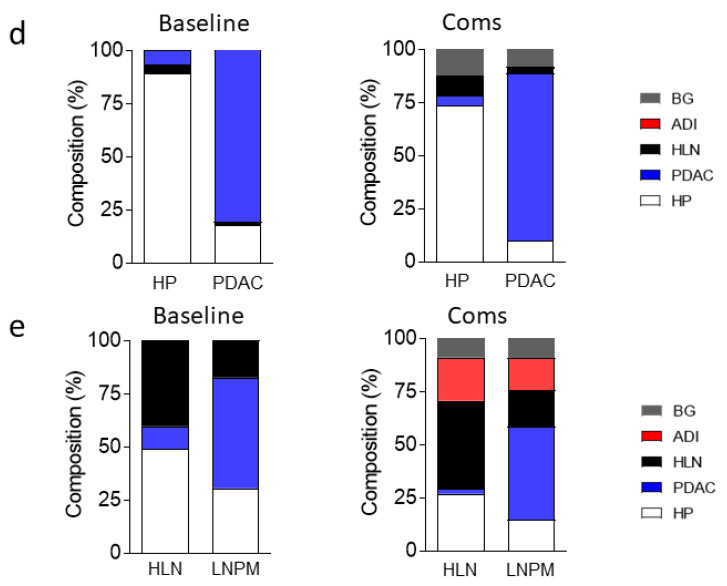
Communicator-based clean-up can be transferred to CNNs using a different input size. (**a**) Selection of the normalized 299 × 299 × 3 tissue patches based on the classification of the communicator CNNs using a 224 × 224 × 3 crop of the image tiles is illustrated (Spot: Scale bar = 300 μm, Patch: Scale bar = 60 μm). (**b**) Heatmap of the performance difference between the inceptionnetv3 trained on data with and without the data clean-up via the communicators is shown. (**c**) Visualization of the classified validation data by the baseline or cleaned-up inceptionv3 net. Healthy lymph node (HLN, yellow), healthy pancreas (HP, red), pancreatic ductal adenocarcinoma (PDAC, blue), background (BG, grey) and adipose tissue (ADI, cyan) classification is illustrated (scale bar = 2 mm). (**d**) Pooled classification, as determined using a baseline and cleaned inceptionv3 network from healthy pancreas (HP) (*n* = 3) and pancreatic ductal adenocarcinoma (PDAC) images (*n* = 15), is illustrated. (**e**) Average of classification, as determined using a baseline and cleaned inceptionv3 network of images showing healthy lymph nodes (HLN) (*n* = 5) and lymph nodes with pancreatic ductal adenocarcinoma metastasis (LNPM) (*n* = 6), is presented (ADI= adipose tissue, BG = background).

Next, we wondered whether we could use the data preprocessing to purify the dataset for CNNs using another input size. We hypothesized that by using the clean-up process with the 224 × 224 × 3 labeled image dataset, we could extract a cleaned 299 × 299 × 3 image tile dataset needed to train an inceptionv3 CNN [33]. Specifically, we mapped the 299 × 299 × 3 image tiles and classified a cropped section (224 × 224 × 3) via the communicators (Figure 7a). The labels were transferred to normalized image tiles to establish an improved ground truth (Figure 7a). The performance of the cleaned-up inceptionv3 CNN was increased compared to the baseline model (Figure 7b, Supplementary Table S3). Furthermore, the communicator preprocessed network was able to better label the independent validation dataset when compared to the baseline model (Figure 7c–e). These data indicate that a communicator-based clean-up process can potentially be transferred to CNNs with unmatching input sizes.

## 4. Discussion

In the current investigation, we show that PDAC can be detected with the help of convolutional neural networks using deep transfer learning. We introduced a dataset preprocessing step to purify dataset classes according to new labels via two communicators. As a result of this purification step, we increased the ground truth and, therefore, the performance of image classification on an independent validation dataset. Furthermore, we titrated several networks and hyperparameters to optimize their performance.

In daily diagnostic practice, carcinomas are classified on the basis of their characteristic histomorphology and immunohistochemical marker profiles. While different cancer types can be distinguished by deep learning algorithms based on data retrieved from the Cancer Genome Atlas [14], the diagnosis of PDAC metastases can be challenging due to overlapping features with other entities, such as biliary cancer. Here, we show that, based on a dataset of 460 tissue spots (223 PDAC, 161HP, 76 HLN), tissue entities could be correctly labeled in images from independent tissue sections. The short time taken to classify an image might be useful to potentially aid pathologists during tissue evaluation. If several cases/slides have to be evaluated, the algorithm could potentially be employed to highlight areas of interest for the pathologist. This could be achieved, for example, by annotating the cases/slides and/or by flagging unclear cases. For example, the algorithm could flag areas of interest (i.e., areas of suspected cancer infiltration, e.g., in lymph nodes) that should be examined first by the pathologist. It is also imaginable that the algorithm could be exploited to aid pathologists with measurements (e.g., measuring the diameter of tumor formations, or measuring distances from the tumor to resection margins). However, it remains essential that a trained pathologist examines histopathological images and makes decisions involving the diagnosis, treatment regimens, and prognosis. Deep-learning-based algorithms carry the risk of methodical biases, such as overfitting, imperfect ground truth, variation in reproducible staining patterns, and confusion with untrained tissue types. Moreover, installation costs, such as histological slide digitalization and computational capacities, apply, although, overall, the use of machine learning algorithms is cost-effective. In the current state, our algorithm and the underlying program needs further development before being potentially applied for clinical use. Future development using data from large multicentered cohorts with solid labeled ground truths might improve CNNs in their role to help in the classification and quantification of histopathological images. The question of whether the described communicator approach can help with establishing a ground truth also in other datasets, including for different cancer types, needs more exploration. For a training dataset with more class labels, for example, a cancer-associated stroma or inflamed/necrotic tissue, the clean-up process could be potentially further improved. Although biopsy samples enable pathologists to make a definite diagnosis in most cases, contexts in which a primary tumor cannot be determined are known to exist both in PDAC diagnostics and in the diagnostics of other tumors [34]. Therefore, future studies should also focus on where the gaps are and which type of diagnostic-setting deep-learning-based algorithms can best be used to maximize its utility. Furthermore, whether the communicator approach can be used for other cancer identities or detect cancer tissue in different organs needs to be further evaluated. In principle, the data clean-up procedure can be transferred to different tasks. However, whether other cancer types can benefit from the use of communicator-based pre-processing needs to be shown for individual datasets to support this speculation.

Importantly, we demonstrate the ability to correctly classify image tiles derived from healthy or metastatic lymph node tissues. However, we also observed a proportion of mislabeled image tiles in these datasets. Specifically, these areas showed other tissue types, such as vasculature, which caused confusion in the labeling network. This indicates that further datasets are required to increase the performance of neural networks and that therapeutic decisions, ultimately, are dependent on the physicians.

Dataset purification can improve the performance of convolutional neural networks. Digital pathology can assist pathologists with classifying histopathological images [35]. These networks are trained on large datasets from various public sources, including PubMed and The Cancer Genome Atlas [14,35]. However, automated software-supported analysis of histological slides is often hampered by the presence of different tissue types on the histology slide. Hence, dataset preprocessing can help to increase the quality of the ground truth. In this study, we used an existing dataset containing adipose tissue to eliminate tissue tiles from our new dataset [10]. This was performed using two communicators, which cleaned up the dataset in cycles. The purified dataset could improve the performance of the convolutional neural network. This automated process might be useful to identify and label pathologic tissue identities. The correct identification of adipose tissue in particular is an important aspect of the deep-learning-based analysis of histological slides. Locally advanced invasive cancer will often infiltrate organ-surrounding adipose tissue. In order to use deep-learning-based analyses of histologic slides to determine classical prognostic parameters, such as the tumor diameter or the minimal distance of the tumor to the resection margins, a precise distinction between tumor tissue and adipose tissue is crucial. This distinction between tumor and fatty tissue is also important in the detection of the extracapsular extension of lymph node metastases into the lymph-node-surrounding adipose tissue, which has been shown to be a prognostic factor in various solid cancers [36,37,38]. Other, more experimental approaches, such as the detection of so-called Stroma AReactive Invasion Front Areas (SARIFA) as a potential prognostic factor in gastrointestinal cancers, also strongly depend on the distinction between the tumor’s invasive front and its inconspicuous surrounding fatty tissue [39]. Whether deep-learning-based algorithms and the communicator-based approach can be successfully used to aid in the distinction between adipose and tumor tissue remains to be determined.

Hyperparameter tuning can determine the performance of neural networks. A variety of convolutional neural networks are used to analyze histological images. Specifically, a ResNet-50 architecture was used to classify large histological datasets [35]. Furthermore, other architectures, including GoogLeNet, AlexNet, and Vgg-16, were successfully used for classifying histopathological images [40]. Since all these network architectures share the same input size of 224 × 224, our hyperparameter tuning was focused on these models. Our data show that several convolutional neural networks were able to distinguish between PDAC, healthy lymph nodes, adipose tissue and healthy pancreas tissue. However, when we tested several networks and the hyperparameters during training, we found that VGG19 with a learning rate of 10^−5^ and ADAM as an optimizer was ideal for our task. Future studies should investigate whether these differences are task specific. Notably, the use of inception v3, which performed very well in other tasks using H&E tissue sections [8,14], relies on an input data size of 299 × 299. Using the communicator approach, the ground truth was improved by transferring the image tile classification of the communicators to 299 × 299 image tiles.

## 5. Conclusions

In conclusion, our study shows that dataset preprocessing via two communicators and hyperparameter tuning can improve classification performance to identify PDAC on H&E tissue sections. Further studies applying this approach to metastases from different primaries are needed for validation.

## Figures and Tables

**Table 1:** Metrics of the uncleaned network (ResNet18): Precision, Recall, F1-Score and Jaccard score for the classes healthy pancreas (HP), healthy lymph node (HLN) and pancreatic ductal adenocarcinoma (PDAC).

Class	Precision	Recall	F1-Score	Jaccard Score	Support
HLN	0.89	0.91	0.9	0.82	874
HP	0.9	0.82	0.86	0.75	1690
PDAC	0.9	0.94	0.92	0.85	2454
Accuracy			0.9		5018
Macro avg	0.9	0.89	0.89	0.81	5018
Weighted avg	0.9	0.9	0.9	0.81	5018

**Table 2:** Metrics of the cleaned network (ResNet18): Precision, Recall, F1-Score and Jaccard score for the classes healthy pancreas (HP), healthy lymph node (HLN), pancreatic ductal adenocarcinoma (PDAC) and Adipose tissue (ADI).

Class	Precision	Recall	F1-Score	Jaccard	Support
ADI	0.78	0.55	0.64	0.47	51
BG	0.95	0.96	0.96	0.92	908
HLN	0.92	0.94	0.93	0.87	758
HP	0.97	0.91	0.94	0.88	1277
PDAC	0.93	0.96	0.94	0.89	1910
Accuracy			0.94		4904
Macro avg	0.91	0.86	0.88	0.81	4904
Weighted avg	0.94	0.94	0.94	0.89	4904

## Data Availability

The source code is available at: https://github.com/MolecularMedicine2/pypdac (accessed on 13 March 2022).

## Supplementary Materials

The following supporting information can be downloaded at: https://www.mdpi.com/article/10.3390/cancers14081964/s1: Supplementary Figure S1: Tissue Micro Arrays enable staining and presentation of multiple patient tissue sections on one histological slide.; Supplementary Figure S2: Percentage of discarded image patches of the different tissue types during the cleanup process; Supplementary Figure S3: ComCylce3 on the extern validation data shows improvement with only 3 cycles; Supplementary Figure S4: Baseline and Coms with Pixelcutoff instead of background class. Coms still outperform Baseline on the extern validation data with *n* = 10 Cycles for the Communicators; Supplementary Figure S5: Receiver operating characteristic for the different tissue classes; Supplementary Table S1: Patients Data; Supplementary Table S2: Differences of the metrics of the cleaned and uncleaned network; Supplementary Table S3: Overview of the seven CNN configuration used for the experiments; Supplementary Table S4: Network parameters and metrics from the 72 nets from Hyperparameter-Tuning are shown.
