# Supplementary material for: Communicator-Driven Data Preprocessing Improves Deep Transfer Learning of Histopathological Prediction of Pancreatic Ductal Adenocarcinoma
Source: Cancers (Basel). 2022 Apr 13;14(8):1964. doi: 10.3390/cancers14081964 (PMC9031738; doi:10.3390/cancers14081964)
Supplement: Supplementary file 1 [file cancers-14-01964-s001.zip › cancers-1658352-supplementary.pdf]

Supplementary Figure S1

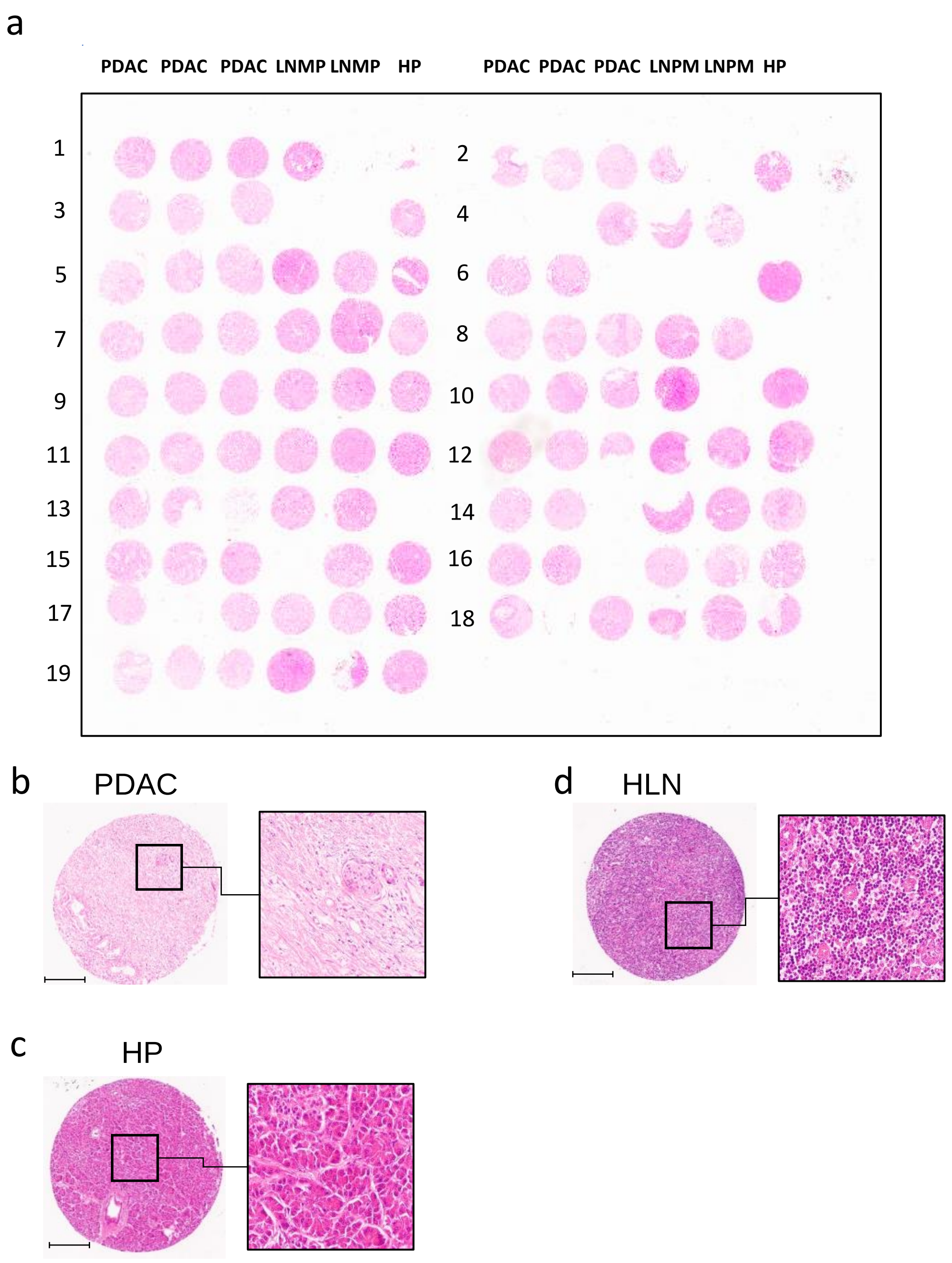

Supplementary Figure S1: Tissue Micro Arrays enable staining and presentation of multiple patient tissue sections on one histological slide. (a) TMA with three spots of pancreatic ductal adenocarcinoma (PDAC), two lymph nodes with metastasis from pancreatic ductal adenocarcinoma (LNPM) and one healthy pancreas (HP) per patient are shown. Healthy lymph nodes are on different TMAs. Representative images and zoom from H&E-stained samples of **(b)** pancreatic ductal adenocarcinoma (PDAC), **(c)** healthy pancreas (HP) and **(d)** healthy lymph node (HLN) are shown (Scalebar = 300 µm).

Supplementary Figure S2

a

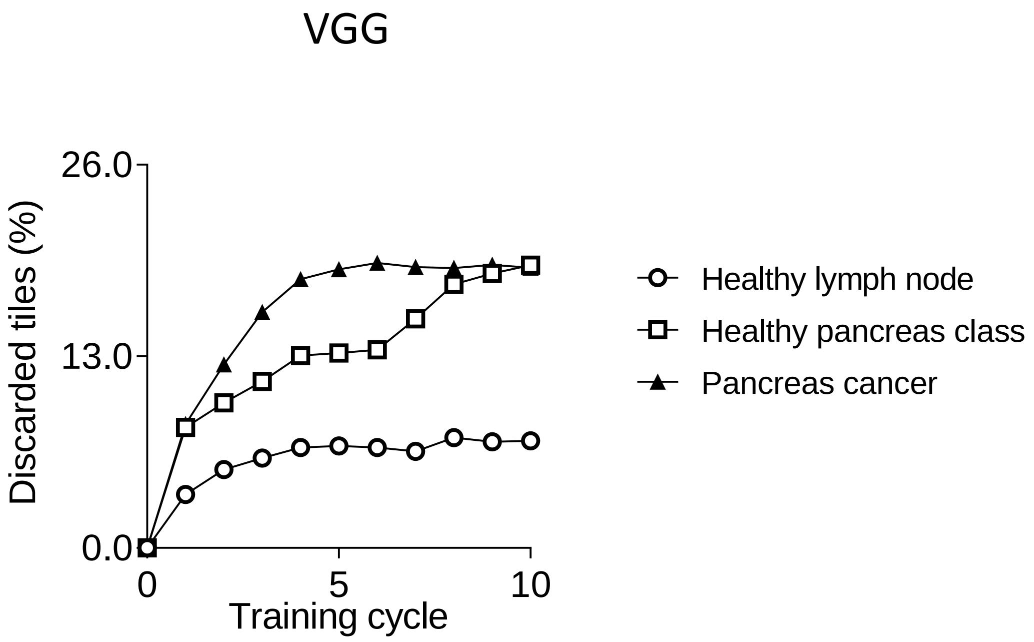

b

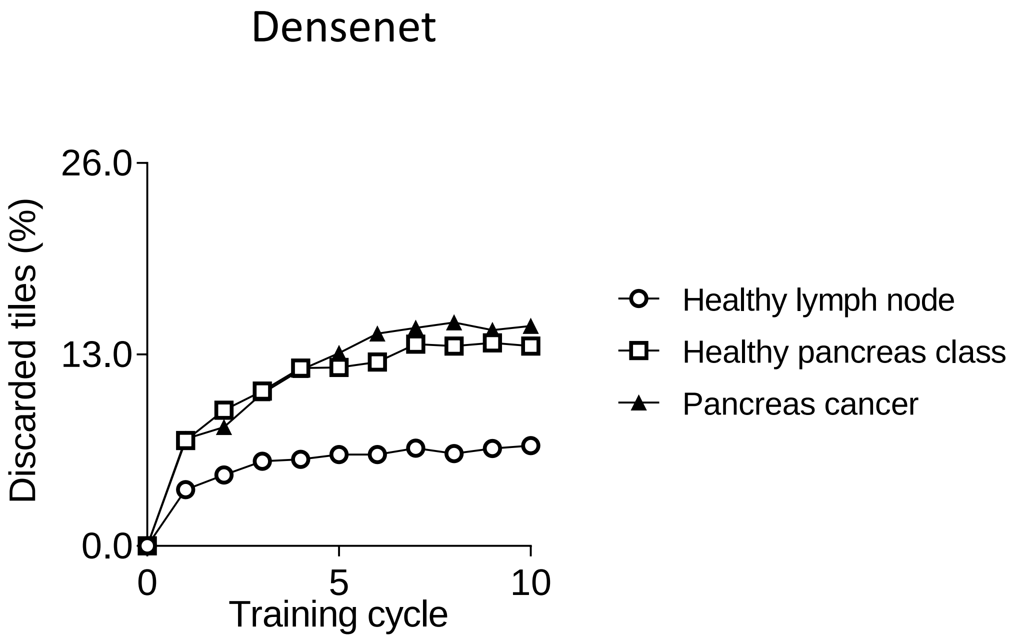

Supplementary Figure S2: Percentage of discarded image patches of the different tissue types during the cleanup process from healthy lymph nodes, healthy pancreas and pancreatic ductal adenocarcinoma is indicated for **(a)** VGG and **(b)** Densenet are shown.

Supplementary Figure S3

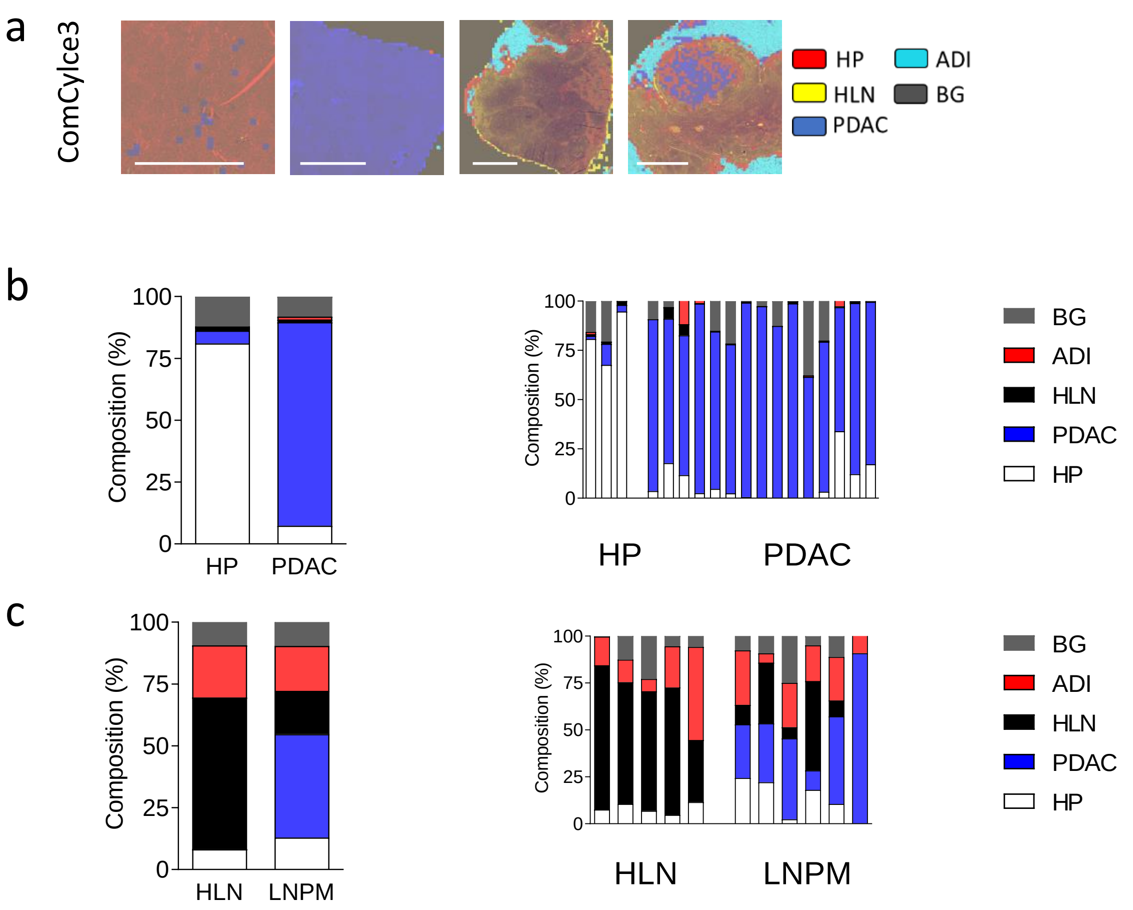

Supplementary Figure S3: ComCylce3 on the extern validation data shows improvement with only 3 cycles. Colored external validation images with the **(a)** Baseline model and with the Cutoff Communicators model are shown. **(b)** Pooled and Individual classification as determined using an cutoff baseline and cutoff cleaned of whole images slides from healthy pancreas (HP) (n=3) and pancreatic ductal adenocarcinoma (PDAC) (n=15) **(c)** Pooled and Individual classification as determined using an cutoff baseline and cutoff cleaned network of whole images slides from healthy lymph nodes (HLN) (n=5) and lymph nodes with metastasis from pancreatic ductal adenocarcinoma (LNPM) (n=6) are shown.

Supplementary Figure S4

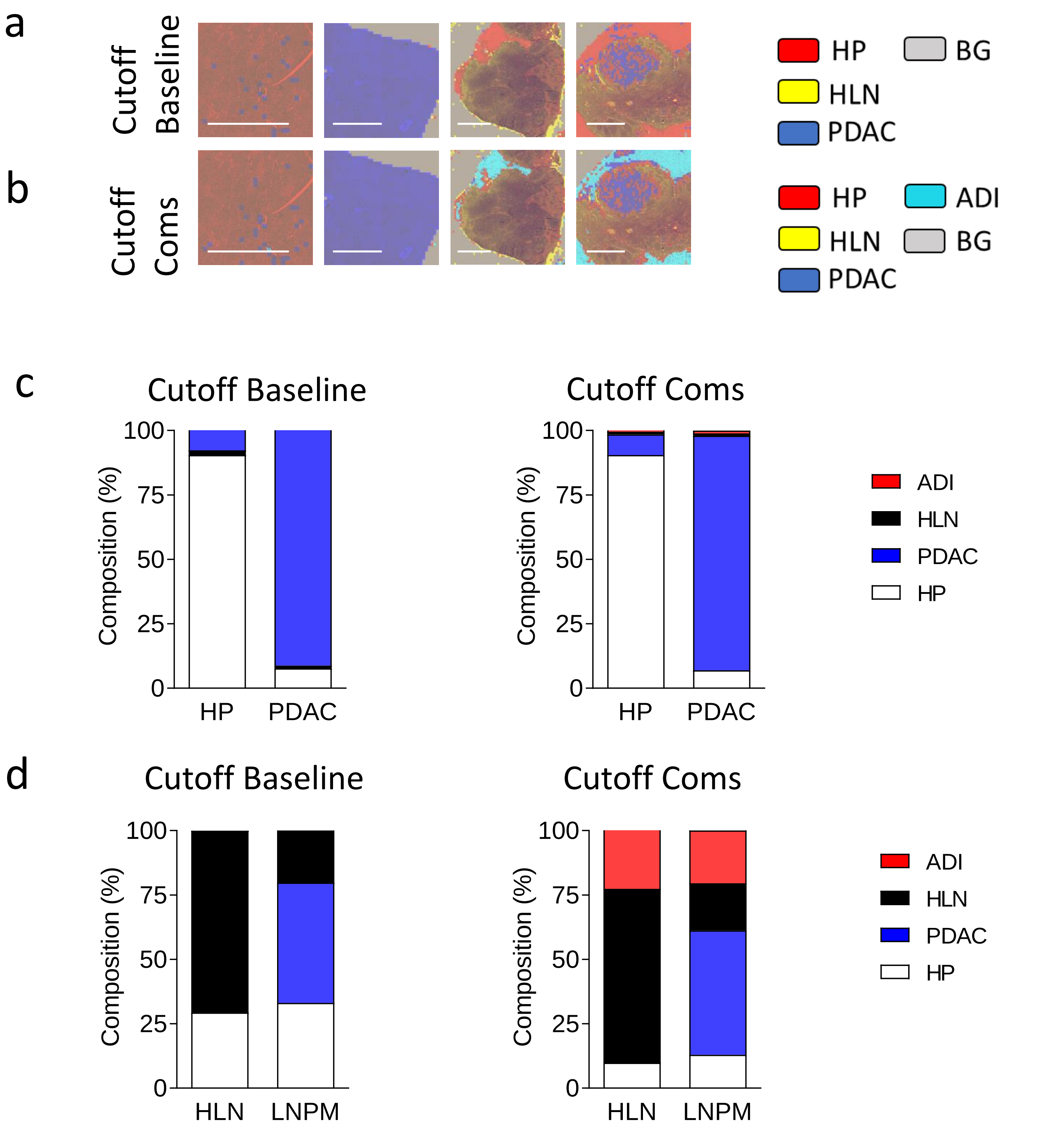

Supplementary Figure S4: Baseline and Coms with Pixelcutoff instead of background class. Coms still outperform Baseline on the extern validation data with n =10 Cycles for the Communicators. Colored external validation images with the **(a)** Cutoff Baseline model and **(b)** with the Cutoff Communicators model are shown. **(c)** Pooled classification as determined using an cutoff baseline and cutoff cleaned of whole images slides from healthy pancreas (HP) (n=3) and pancreatic ductal adenocarcinoma (PDAC) (n=15) are shown. **(d)** Pooled classification as determined using an cutoff baseline and cutoff cleaned network of whole images slides from healthy lymph nodes (HLN) (n=5) and lymph nodes with metastasis from pancreatic ductal adenocarcinoma (LNPM) (n=6) are shown.

Supplementary Figure S5

a

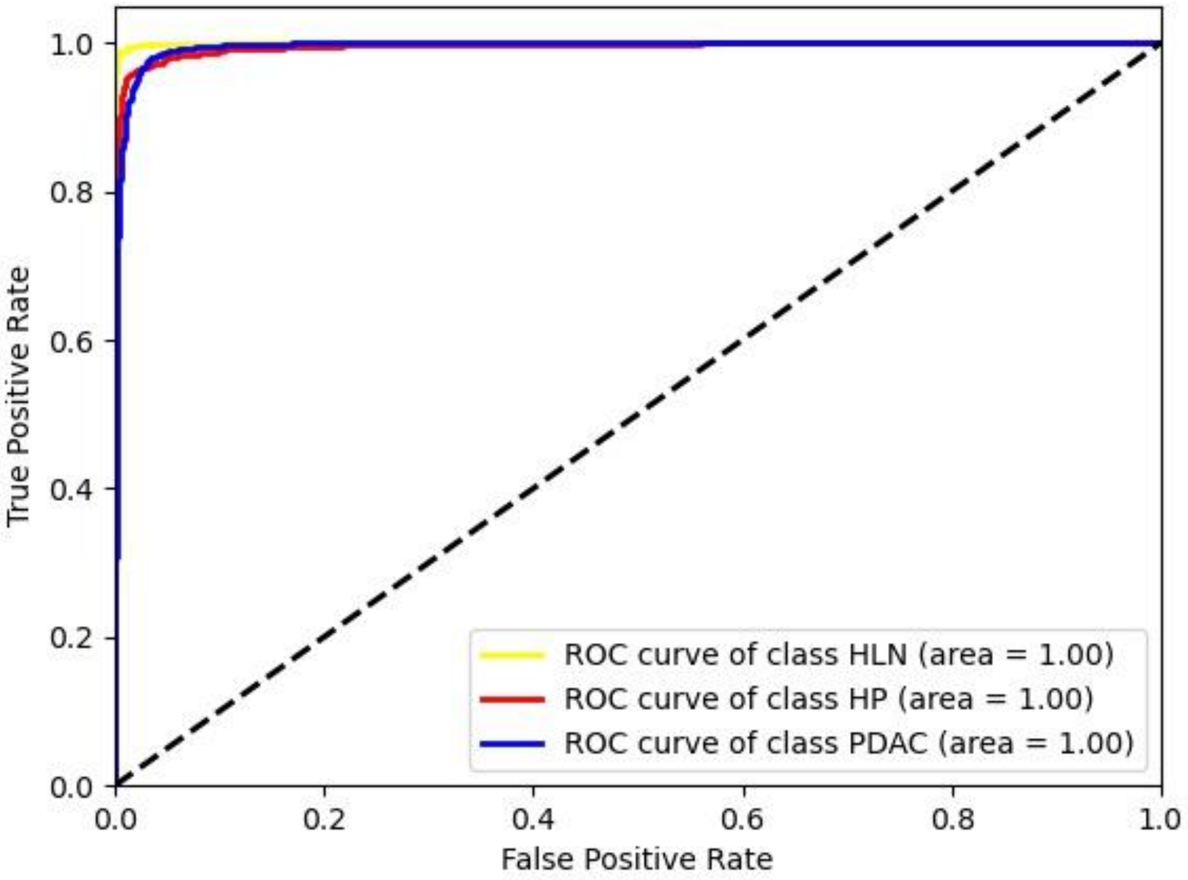

b

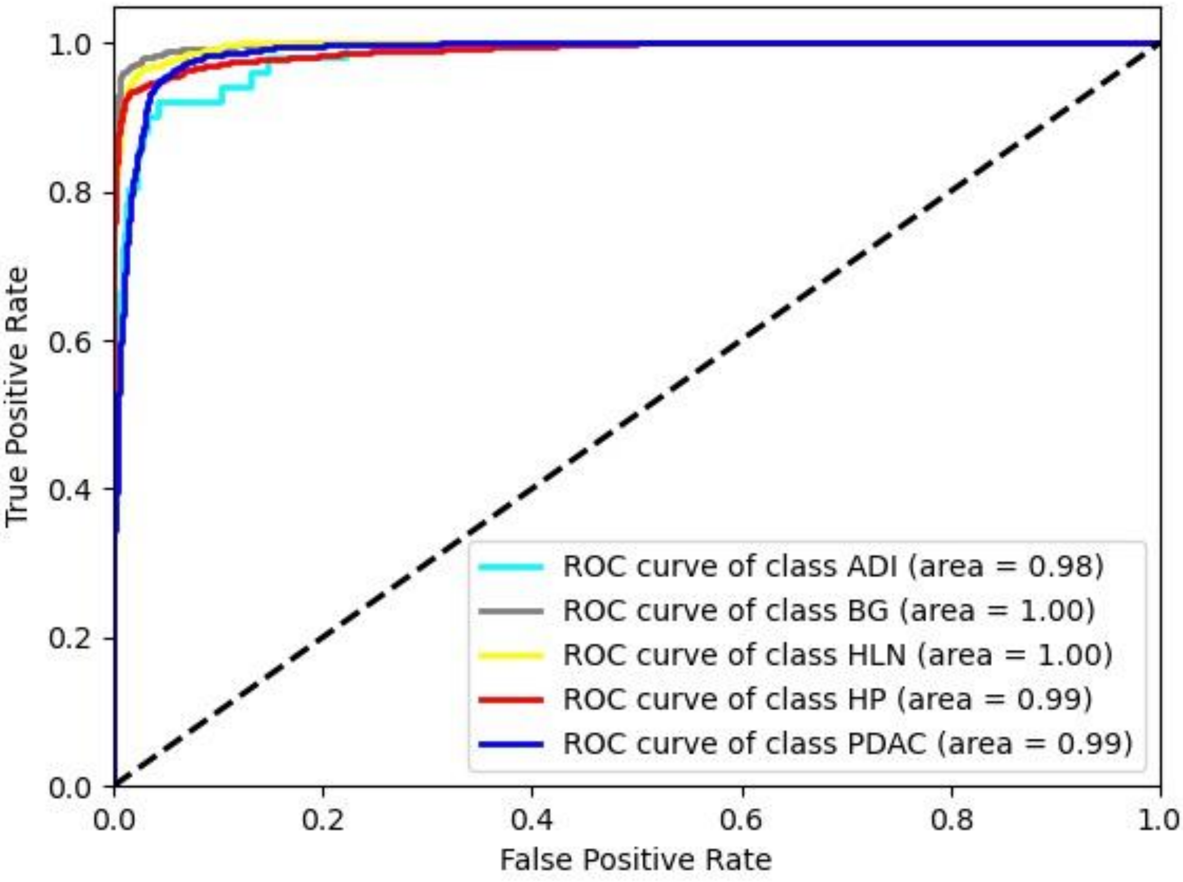

Supplementary Figure S5: Receiver operating characteristic for the different tissue classes for **(a)** Baseline evaluated on the cleaned dataset and for the **(b)** Cleaned network evaluated on the cleaned dataset are shown.

Supplementary Table S1

class	Sex (male(%)/female(%))	Median Age (range)	Number of Spots	Number of patients
**PDAC**	52.9 / 47.1	68 (41-90)	223	223
**HLN**	anonym	anonym	76	78
**HP**	52.9 / 47.1	68 (41-90)	161	164

Supplementary Table S1: Patients Data: Age, Gender and Number of Spots for the three classes healthy pancreas (HP), healthy lymph node (HLN) and Pancreatic ductal adenocarcinoma (PDAC) are provided.

Supplementary Data

Supplementary Table S2

class	precision	recall	f1-score	jaccard-score
**HLN**	0.03	0.03	0.07	0.05
**HP**	0.07	0.09	0.11	0.13
**PDAC**	0.03	0.02	0.05	0.04
**accuracy**			0.04	
**macro avg**	0.01	-0.03	-0.01	0
**weighted avg**	0.04	0.04	0.04	0.08

Supplementary Table S2: Differences of the metrics of the cleaned and uncleaned network: Accuracy, Precision, Recall, F1-Score and Jaccard score for the classes healthy pancreas (HP), healthy lymph node (HLN) and Pancreatic ductal adenocarcinoma (PDAC) are shown.

Supplementary Table S3

Name	Model	Patchsize	Batchsize	Optimizer	Learning rate	Cutoff	Com Cycles
Baseline	resnet18	224x224x3	150	ADAM	0.0001	No	-
Cleaned network	resnet18	224x224x3	150	ADAM	0.0001	No	10
Inception Baseline	inception	299x299x3	75	RMSprop	0.01	No	-
Inception Coms	inception	299x299x3	75	RMSprop	0.01	No	10
Cutoff Baseline	resnet18	224x224x3	150	ADAM	0.0001	239	-
Cutoff Coms	resnet18	224x224x3	150	ADAM	0.0001	239	10
ComCylce3	resnet18	224x224x3	150	ADAM	0.0001	No	3

Supplementary Table S3: Overview of the seven CNN configuration used for the experiments. (Excluding the models from hyperparamter tuning.)

Supplementary Table S4

model_pre	model_name	lr	opt	balanced accuracy	precision	recall	f1 score	jaccard score	roc_auc_score	HLN score	PDAC score	HP score	LNPM score	test HLN	test PDAC	test HP	four_score	three_score
alexnet_1	alexnet	0.0001	ADAM	0.857	0.944	0.945	0.944	0.896	0.994	0.969	0.89	0.978	0.997	0.945	0.959	0.928	0.958	0.944
alexnet_2	alexnet	0.0001	SGD	0.857	0.945	0.945	0.944	0.897	0.994	0.968	0.898	0.979	0.997	0.945	0.962	0.924	0.96	0.944
alexnet_3	alexnet	0.0001	RMSprop	0.831	0.937	0.937	0.935	0.881	0.988	0.944	0.942	0.985	0.989	0.943	0.965	0.898	0.965	0.935
alexnet_4	alexnet	0.00001	ADAM	0.801	0.92	0.921	0.92	0.855	0.987	0.962	0.851	0.979	0.991	0.918	0.939	0.894	0.946	0.917
alexnet_5	alexnet	0.00001	SGD	0.797	0.919	0.92	0.918	0.853	0.987	0.963	0.842	0.978	0.989	0.918	0.935	0.896	0.943	0.916
alexnet_6	alexnet	0.00001	RMSprop	0.823	0.929	0.93	0.929	0.869	0.99	0.959	0.858	0.977	0.988	0.927	0.942	0.911	0.946	0.927
alexnet_7	alexnet	0.000001	ADAM	0.666	0.824	0.83	0.825	0.71	0.955	0.894	0.855	0.997	0.883	0.71	0.853	0.809	0.907	0.791
alexnet_8	alexnet	0.000001	SGD	0.666	0.824	0.83	0.825	0.71	0.955	0.894	0.855	0.997	0.883	0.71	0.853	0.809	0.907	0.791
alexnet_9	alexnet	0.000001	RMSprop	0.691	0.847	0.856	0.851	0.747	0.965	0.95	0.881	0.995	0.866	0.805	0.878	0.814	0.923	0.832
densenet_1	densenet	0.0001	ADAM	0.875	0.96	0.961	0.96	0.925	0.997	0.988	0.891	0.978	0.995	0.985	0.968	0.955	0.963	0.969
densenet_2	densenet	0.0001	SGD	0.876	0.962	0.962	0.961	0.927	0.997	0.987	0.895	0.977	0.996	0.987	0.97	0.956	0.964	0.971
densenet_3	densenet	0.0001	RMSprop	0.877	0.964	0.964	0.963	0.931	0.997	0.982	0.861	0.97	0.996	0.984	0.975	0.952	0.952	0.97
densenet_4	densenet	0.00001	ADAM	0.773	0.928	0.926	0.923	0.862	0.986	0.984	0.939	0.992	0.911	0.914	0.952	0.905	0.956	0.924
densenet_5	densenet	0.00001	SGD	0.773	0.928	0.926	0.923	0.862	0.986	0.984	0.939	0.992	0.911	0.914	0.951	0.905	0.956	0.923
densenet_6	densenet	0.00001	RMSprop	0.82	0.938	0.937	0.936	0.882	0.99	0.989	0.947	0.992	0.959	0.938	0.962	0.913	0.972	0.938
densenet_7	densenet	0.000001	ADAM	0.649	0.794	0.629	0.668	0.523	0.905	0.901	0.457	0.951	0.86	0.679	0.363	0.793	0.792	0.612
densenet_8	densenet	0.000001	SGD	0.649	0.794	0.629	0.668	0.523	0.905	0.901	0.457	0.951	0.86	0.679	0.363	0.793	0.792	0.612
densenet_9	densenet	0.000001	RMSprop	0.729	0.839	0.765	0.791	0.662	0.939	0.934	0.714	0.99	0.986	0.782	0.635	0.841	0.906	0.753
resnet_1	resnet	0.0001	ADAM	0.877	0.947	0.947	0.947	0.9	0.994	0.99	0.942	0.998	0.981	0.946	0.962	0.93	0.978	0.946
resnet_2	resnet	0.0001	SGD	0.877	0.947	0.947	0.947	0.9	0.994	0.99	0.942	0.998	0.981	0.946	0.962	0.93	0.978	0.946
resnet_3	resnet	0.0001	RMSprop	0.871	0.948	0.949	0.948	0.903	0.995	0.98	0.933	0.999	0.981	0.95	0.963	0.935	0.973	0.949
resnet_4	resnet	0.00001	ADAM	0.792	0.917	0.916	0.914	0.844	0.983	0.944	0.963	0.97	0.962	0.906	0.939	0.878	0.96	0.908
resnet_5	resnet	0.00001	SGD	0.792	0.917	0.916	0.914	0.844	0.983	0.944	0.963	0.97	0.962	0.906	0.939	0.878	0.96	0.908
resnet_6	resnet	0.00001	RMSprop	0.824	0.927	0.927	0.926	0.864	0.987	0.958	0.958	0.983	0.986	0.921	0.949	0.894	0.971	0.921
resnet_7	resnet	0.000001	ADAM	0.506	0.705	0.659	0.641	0.489	0.899	0.402	0.892	0.6	0.994	0.203	0.735	0.616	0.722	0.518
resnet_8	resnet	0.000001	SGD	0.506	0.705	0.659	0.641	0.489	0.899	0.402	0.892	0.6	0.994	0.203	0.735	0.616	0.722	0.518
resnet_9	resnet	0.000001	RMSprop	0.619	0.787	0.782	0.775	0.64	0.937	0.518	0.954	0.796	0.932	0.545	0.829	0.748	0.8	0.707
resnet101_1	resnet101	0.0001	ADAM	0.872	0.951	0.951	0.951	0.908	0.994	0.992	0.849	0.982	0.938	0.971	0.953	0.949	0.94	0.958
resnet101_2	resnet101	0.0001	SGD	0.872	0.951	0.952	0.951	0.909	0.994	0.992	0.849	0.981	0.938	0.971	0.954	0.95	0.94	0.958
resnet101_3	resnet101	0.0001	RMSprop	0.861	0.951	0.951	0.951	0.909	0.994	0.999	0.869	0.996	0.944	0.976	0.958	0.943	0.952	0.959
resnet101_4	resnet101	0.00001	ADAM	0.794	0.932	0.93	0.928	0.869	0.989	0.824	0.923	0.987	0.964	0.931	0.964	0.895	0.924	0.93
resnet101_5	resnet101	0.00001	SGD	0.794	0.932	0.93	0.928	0.869	0.989	0.824	0.923	0.987	0.964	0.931	0.964	0.895	0.924	0.93
resnet101_6	resnet101	0.00001	RMSprop	0.833	0.933	0.933	0.932	0.875	0.991	0.903	0.89	0.989	0.939	0.943	0.954	0.906	0.93	0.934
resnet101_7	resnet101	0.000001	ADAM	0.624	0.764	0.681	0.675	0.522	0.942	0.68	0.551	0.976	0.675	0.715	0.438	0.847	0.72	0.667
resnet101_8	resnet101	0.000001	SGD	0.624	0.764	0.681	0.675	0.522	0.942	0.68	0.551	0.976	0.675	0.715	0.438	0.847	0.72	0.667
resnet101_9	resnet101	0.000001	RMSprop	0.694	0.843	0.842	0.839	0.729	0.959	0.644	0.853	0.989	0.974	0.794	0.808	0.865	0.865	0.822
resnet50_1	resnet50	0.0001	ADAM	0.874	0.952	0.952	0.952	0.91	0.996	0.985	0.926	0.984	0.982	0.97	0.972	0.93	0.969	0.957
resnet50_2	resnet50	0.0001	SGD	0.866	0.951	0.951	0.951	0.908	0.996	0.986	0.93	0.984	0.976	0.971	0.966	0.939	0.969	0.959
resnet50_3	resnet50	0.0001	RMSprop	0.872	0.955	0.956	0.955	0.916	0.996	0.999	0.923	0.981	0.968	0.968	0.974	0.945	0.968	0.962
resnet50_4	resnet50	0.00001	ADAM	0.792	0.937	0.935	0.933	0.878	0.988	0.903	0.973	0.993	0.975	0.943	0.961	0.909	0.961	0.938
resnet50_5	resnet50	0.00001	SGD	0.792	0.937	0.935	0.933	0.878	0.988	0.903	0.973	0.993	0.975	0.943	0.961	0.909	0.961	0.938
resnet50_6	resnet50	0.00001	RMSprop	0.827	0.94	0.94	0.939	0.887	0.991	0.937	0.965	0.993	0.999	0.954	0.962	0.914	0.974	0.943
resnet50_7	resnet50	0.000001	ADAM	0.611	0.808	0.796	0.782	0.653	0.944	0.626	0.979	0.826	0.765	0.475	0.949	0.679	0.799	0.701
resnet50_8	resnet50	0.000001	SGD	0.611	0.808	0.796	0.782	0.653	0.944	0.626	0.979	0.826	0.765	0.475	0.949	0.679	0.799	0.701
resnet50_9	resnet50	0.000001	RMSprop	0.674	0.859	0.855	0.848	0.743	0.958	0.756	0.982	0.901	0.779	0.701	0.955	0.76	0.854	0.805
squeezenet_1	squeezenet	0.0001	ADAM	0.833	0.933	0.933	0.931	0.874	0.99	0.953	0.945	0.988	0.996	0.938	0.954	0.895	0.97	0.929
squeezenet_2	squeezenet	0.0001	SGD	0.832	0.932	0.932	0.931	0.873	0.99	0.952	0.947	0.988	0.999	0.937	0.954	0.894	0.972	0.928
squeezenet_3	squeezenet	0.0001	RMSprop	0.803	0.924	0.925	0.923	0.861	0.987	0.999	0.943	0.993	0.945	0.927	0.949	0.887	0.97	0.921
squeezenet_4	squeezenet	0.00001	ADAM	0.768	0.895	0.888	0.89	0.807	0.971	0.973	0.959	0.941	0.938	0.877	0.907	0.846	0.953	0.877
squeezenet_5	squeezenet	0.00001	SGD	0.768	0.895	0.889	0.89	0.807	0.971	0.973	0.959	0.941	0.938	0.877	0.907	0.846	0.953	0.877
squeezenet_6	squeezenet	0.00001	RMSprop	0.784	0.904	0.902	0.902	0.825	0.977	0.969	0.959	0.944	0.978	0.88	0.931	0.856	0.962	0.889
squeezenet_7	squeezenet	0.000001	ADAM	0.342	0.453	0.47	0.454	0.321	0.665	0.384	0.727	0.635	0.701	0.005	0.666	0.324	0.612	0.332
squeezenet_8	squeezenet	0.000001	SGD	0.343	0.453	0.471	0.454	0.321	0.665	0.384	0.727	0.635	0.701	0.005	0.666	0.325	0.612	0.332
squeezenet_9	squeezenet	0.000001	RMSprop	0.464	0.597	0.626	0.602	0.466	0.801	0.428	0.854	0.696	0.634	0.09	0.77	0.555	0.653	0.472
vgg16_1	vgg16	0.0001	ADAM	0.884	0.957	0.957	0.957	0.919	0.997	0.987	0.797	0.971	0.952	0.963	0.953	0.967	0.927	0.961
vgg16_2	vgg16	0.0001	SGD	0.881	0.957	0.958	0.957	0.92	0.997	0.988	0.811	0.973	0.96	0.963	0.954	0.966	0.933	0.961
vgg16_3	vgg16	0.0001	RMSprop	0.871	0.959	0.96	0.959	0.924	0.997	0.986	0.818	0.972	0.939	0.971	0.963	0.96	0.929	0.965
vgg16_4	vgg16	0.00001	ADAM	0.859	0.939	0.94	0.939	0.887	0.992	0.991	0.823	0.974	0.99	0.96	0.948	0.922	0.945	0.943
vgg16_5	vgg16	0.00001	SGD	0.858	0.939	0.939	0.939	0.886	0.992	0.992	0.823	0.974	0.99	0.96	0.948	0.922	0.945	0.943
vgg16_6	vgg16	0.00001	RMSprop	0.872	0.942	0.942	0.942	0.892	0.994	0.989	0.785	0.973	0.974	0.96	0.947	0.93	0.93	0.946
vgg16_7	vgg16	0.000001	ADAM	0.676	0.862	0.856	0.85	0.746	0.961	0.875	0.991	0.949	0.748	0.761	0.979	0.717	0.891	0.819
vgg16_8	vgg16	0.000001	SGD	0.678	0.864	0.858	0.852	0.749	0.962	0.88	0.992	0.952	0.749	0.772	0.979	0.719	0.893	0.823
vgg16_9	vgg16	0.000001	RMSprop	0.72	0.891	0.895	0.89	0.809	0.974	0.951	0.966	0.992	0.805	0.884	0.954	0.814	0.928	0.884
vgg19_1	vgg19	0.0001	ADAM	0.876	0.962	0.962	0.962	0.929	0.997	0.975	0.867	0.978	0.976	0.975	0.97	0.962	0.949	0.969
vgg19_2	vgg19	0.0001	SGD	0.884	0.963	0.963	0.963	0.93	0.997	0.97	0.873	0.98	0.977	0.972	0.971	0.96	0.95	0.968
vgg19_3	vgg19	0.0001	RMSprop	0.886	0.962	0.962	0.962	0.928	0.997	0.98	0.864	0.974	0.96	0.971	0.975	0.954	0.944	0.967
vgg19_4	vgg19	0.00001	ADAM	0.833	0.938	0.938	0.937	0.884	0.993	0.985	0.902	0.986	0.994	0.943	0.952	0.924	0.967	0.94
vgg19_5	vgg19	0.00001	SGD	0.833	0.937	0.938	0.937	0.883	0.993	0.985	0.901	0.986	0.993	0.943	0.952	0.924	0.966	0.94
vgg19_6	vgg19	0.00001	RMSprop	0.837	0.942	0.942	0.941	0.892	0.995	0.971	0.884	0.984	0.997	0.955	0.954	0.93	0.959	0.946
vgg19_7	vgg19	0.000001	ADAM	0.697	0.878	0.881	0.876	0.786	0.969	0.827	0.987	0.954	0.759	0.768	0.96	0.839	0.882	0.856
vgg19_8	vgg19	0.000001	SGD	0.697	0.878	0.881	0.876	0.786	0.969	0.827	0.987	0.954	0.759	0.768	0.96	0.839	0.882	0.856
vgg19_9	vgg19	0.000001	RMSprop	0.722	0.898	0.904	0.899	0.824	0.978	0.915	0.98	0.971	0.763	0.852	0.962	0.863	0.907	0.892

Supplementary Table S4: Network parameters and metrics from the 72 nets from Hyperparameter-Tuning are shown. We kept the following parameters constant for all the networks: batch size =256, epoch =100, early stopping 10, learning rate decay 0.95, learning step schedule = 5, retrain the last three blocks / layers.
